# The importance of interacting climate modes on Australia’s contribution to global carbon cycle extremes

**DOI:** 10.1038/srep23113

**Published:** 2016-03-15

**Authors:** James Cleverly, Derek Eamus, Qunying Luo, Natalia Restrepo Coupe, Natascha Kljun, Xuanlong Ma, Cacilia Ewenz, Longhui Li, Qiang Yu, Alfredo Huete

**Affiliations:** 1 School of Life Sciences, University of Technology Sydney, PO Box 123, Broadway, NSW, 2007, Australia; 2 Australian Supersite Network, Terrestrial Ecosystem Research Network, University of Technology Sydney.; 3 Climate Change Cluster, University of Technology Sydney.; 4 Department of Geography, Swansea University, Singleton Park, Swansea SA2 8PP, UK; 5 Airborne Research Australia, Flinders University, PO Box 335, Salisbury, South Australia, 5106, Australia

## Abstract

The global carbon cycle is highly sensitive to climate-driven fluctuations of precipitation, especially in the Southern Hemisphere. This was clearly manifested by a 20% increase of the global terrestrial C sink in 2011 during the strongest sustained La Niña since 1917. However, inconsistencies exist between El Niño/La Niña (ENSO) cycles and precipitation in the historical record; for example, significant ENSO–precipitation correlations were present in only 31% of the last 100 years, and often absent in wet years. To resolve these inconsistencies, we used an advanced temporal scaling method for identifying interactions amongst three key climate modes (El Niño, the Indian Ocean dipole, and the southern annular mode). When these climate modes synchronised (1999–2012), drought and extreme precipitation were observed across Australia. The interaction amongst these climate modes, more than the effect of any single mode, was associated with large fluctuations in precipitation and productivity. The long-term exposure of vegetation to this arid environment has favoured a resilient flora capable of large fluctuations in photosynthetic productivity and explains why Australia was a major contributor not only to the 2011 global C sink anomaly but also to global reductions in photosynthetic C uptake during the previous decade of drought.

In 2011, one of the most notable events occurred in global water and carbon cycles: oceans levels decreased due to anomalous precipitation over land^1^,^2^, and the associated increase in photosynthetic productivity by terrestrial ecosystems was termed the 2011 global land C sink anomaly^1^,^3^,^4^. Both of these events were associated with the strongest sustained La Niña in over 90 years (since 1917)^1^,^3^. Beginning in 2010 and proceeding through mid-2011, ocean levels reversed a long-term increasing trend by dropping 5 mm, an anomaly that was associated with increased precipitation across the Southern Hemisphere^3^,^5^. This large precipitation pulse persisted as stored water in Australia’s arheic and endorheic basins, which impede runoff to the ocean^1^,^2^. Very large increases in C uptake by terrestrial ecosystems were driven by these extraordinarily wet conditions, especially in Australia, where the majority of the increased net primary productivity (NPP) and reduced fire-induced C emissions (*ca*. 60% of the 2011 global C sink anomaly) occurred in the semi-arid regions^3^.

Our understanding of the importance of the Southern Hemisphere on the global C cycle precedes the 2011 global land C sink anomaly. Earlier in the 21st century (2000–2009), a reduction in global productivity and evapotranspiration was attributed to drought across the Southern Hemisphere, compensating for increased NPP, water-use efficiency and growing-season length in the Northern Hemisphere^6^,^7^,^8^,^9^. Climate-driven forest die-off from drought and warming has become widespread globally^10^, where increased drought and mortality is followed by large reductions of C sinks in forests, grasslands and savannas^11^. However, semi-arid regions in general are particularly susceptible to forest die-off^12^ because ecosystem productivity in semi-arid regions is closely linked to pulses of precipitation^13^,^14^, which experience large fluctuations driven by variations in climate forcing. Vegetation-climate relationships differ amongst continents in the Southern Hemisphere^15^, possibly depending upon the different climate regimes experienced around the globe. In this study, we will focus on the climate modes associated with the contribution of semi-arid regions of Australia to fluctuations in the global C cycle in general, and to the 2011 global land C sink anomaly and preceding droughts in particular.

Weather across Australia is controlled to varying degrees by three ocean-atmosphere systems^2^,^16^, to which we will refer as *climate modes*: El Niño-Southern Oscillation (ENSO, characterised by its extreme phases: El Niño and La Niña) in the tropical Pacific Ocean, the Indian Ocean dipole (IOD) in the tropical Indian Ocean, and the southern annular mode (SAM) in the Southern Ocean. However, it is still not clear how these climate modes interact to produce precipitation patterns. For example, each climate mode contributes individually to the occurrence, not the intensity, of precipitation in eastern Australia^17^. Furthermore, identification of the interactions amongst these climate modes has been slow to emerge. Thus, the connection between IOD and ENSO was still largely unknown before 2008^18^,^19^, although they were theoretically associated^20^,^21^. The strength of the relationships amongst climate modes might shift with time^20^,^22^, which can alternately enhance or reduce the sensitivity of precipitation to climate modes.

We hypothesised that temporal fluctuations in the synchronisation of these climate modes would be associated with extremes of precipitation in central Australia. To test this hypothesis, statistical analyses were resolved by time and timescale using wavelet transformations on the data (see Methods), resulting in a significant advance in the way we conceptualise relationships in time. Understanding climate requires two components^23^: determining correlations and identifying the physical mechanisms responsible for those correlations. The importance of this study is that it identifies the interactions amongst climate modes responsible for correlations with precipitation extremes in Australia and for the consequently large fluctuations in patterns of regional and global productivity. These temporal correlations of climate and precipitation are evaluated within the context of the dominant climate modes in Australia.

## Climate modes

**Figure 1:**
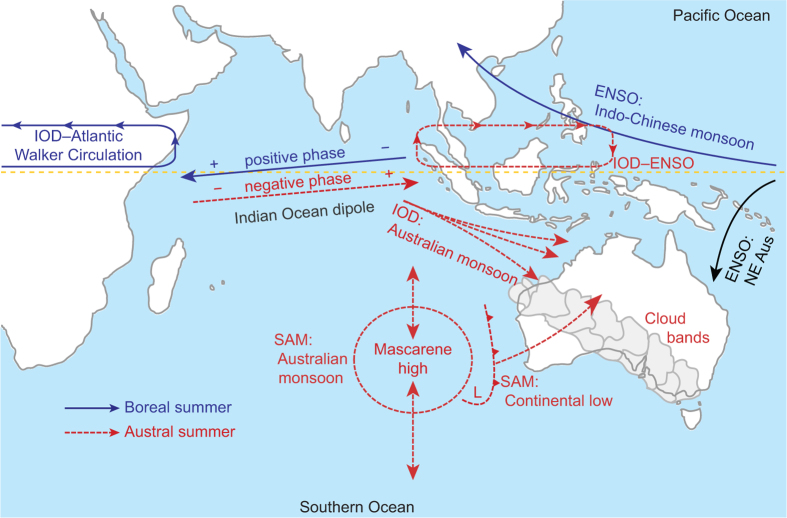
Coupled ocean–climate system in the Indian Ocean region during boreal (solid blue lines) and austral (dashed red lines) summer. Connections between climate modes and weather are indicated with a colon (e.g., ENSO: Indo-Chinese monsoon), and connections amongst climate modes are indicated with a dash (e.g., IOD–ENSO Walker circulation). Arrows near the equator in the Indian Ocean represent development of the convection centre in response to build-up (eastward) and breakdown (westward) of the IOD. See text for further details on the illustrated climate linkages. Map was drawn using Adobe Illustrator CS3 (version 13.0.2, http://www.adobe.com).

Located in the Pacific, the world’s largest ocean, ENSO is the primary driver of global climate and the associated patterns of precipitation, productivity and forest mortality^24^,^25^. ENSO is formed from coupled cross-basin gradients in sea surface temperature (SST) and atmospheric pressure^26^. The warm pole of this gradient is located in the western Pacific Ocean. Whilst the Indo-Chinese monsoon system is directly affected by ENSO (Fig. 1), direct baroclinic effects of ENSO on Australian weather systems are limited to near-tropical eastern Australia^27^, where torrential precipitation and flooding in January 2011 was associated with La Niña^28^. The only influence of ENSO on Australia’s continental weather patterns to the west of the Great Dividing Range (along the east coast) is through the moisture status of the atmosphere, which is maintained *via* Rossby wave trains^25^.

**Figure 2:**
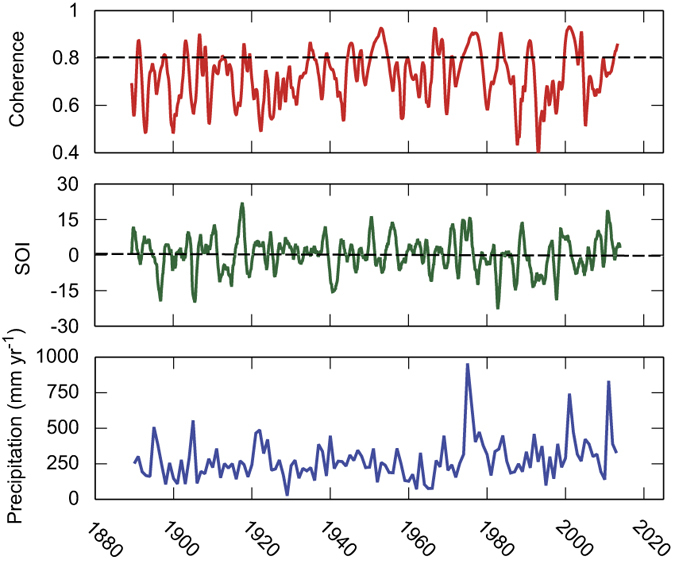
Temporal development of the coherence (squared correlation) between the southern oscillation index (SOI, 12-month moving average) and local precipitation in tropical central Australia (Territory Grape Farm, TGF;Fig. 5). Coherence values above the dashed horizontal line are significantly different from zero (p < 0.05).

To compare long-term trends between ENSO and precipitation in central Australia, we evaluated the temporal correlation using wavelet coherence (i.e., squared correlation resolved in time and frequency space) of the Southern Oscillation index (SOI) *versus* precipitation (Fig. 2). Significant coherence existed between SOI and precipitation, especially during the terrestrial C sink anomaly (2011) and the other very wet years (1976, 2001) in the latter half of the twentieth century (Fig. 2). However, inconsistency in this relationship suggests that ENSO is only indirectly associated with precipitation in central Australia, or this relationship shifts over time due to the intervention of other climate modes. This result is consistent with previous studies in which dry years occurred during normally wet La Niña events and wet years during El Niño^29^.

Like ENSO, IOD is generated by a sea surface temperature (SST) dipole along the equator^30^. In the Indian Ocean, a dipole is established during boreal summer (June–August, Fig. 1), responding to weakening of zonal westerlies, which results in shoaling of the thermocline and cool SST anomalies in the eastern ocean basin (Bjerknes feedback)^19^,^31^. The Australian monsoon depression forms in response to the abrupt breakdown of the IOD during the austral spring (September–November)^20^,^31^. Thus, the strength of the monsoon system in northern Australia (and its ability to penetrate inland) is dictated by the strength of negative IOD anomalies as the convection centre moves eastward across the equatorial Indian Ocean (Fig. 1). Whilst La Niña events occur during extended droughts in southeastern Australia, there is a notable lack of negative IOD events during drought^29^. Similarly, very wet periods in southeastern Australia are often associated with negative IOD events^29^.

SAM represents a north-south oscillatory fluctuation in Southern Ocean SSTs and barometric pressure^32^. The relative strengths of SAM and IOD affect the location of the sub-tropical ridge and associated cloud bands (Fig. 1)^33^. SAM further contributes to modulating the teleconnections between ENSO, IOD and precipitation in Australia^2^ by affecting the position of the Mascarene High over the southern Indian Ocean, which affects the development of the Australian low and the position of the monsoon depression (Fig. 1)^32^,^34^,^35^. The Mascarene High (in the southeast Indian Ocean) forms in response to positive SST anomalies in the northwest Indian Ocean and negative SST anomalies in the southeast^34^. Thus, development of the Mascarene High responds to teleconnections from SAM and from changes in the Atlantic meridional overturning circulation (AMOC) as it leads to re-positioning of the inter-tropical convergence zone (ITCZ) in the tropical Indian Ocean^36^. In contrast to ENSO and IOD, SAM does not appear to have a direct relationship to precipitation in central Australia (Supplementary Fig. 2). Instead, SAM indirectly affects ENSO and IOD *via* the Mascarene High to influence the formation of precipitation extremes throughout the region^35^.

## Synchronisation of climate modes

SOI is represented by the monthly pressure difference between Tahiti and Darwin. The long record of SOI makes it well suited for comparison to historical precipitation, but atmospheric pressure in Darwin can be affected by the state of the Indian Ocean dipole, creating a mixed signal. Similarly, indices that include the Tahiti–Darwin barometric pressure difference (e.g., the multivariate ENSO index, MEI^37^) are likely to be contaminated by IOD-driven trends in atmospheric pressure. In the following analyses, ENSO, IOD and SAM were represented by the Niño3.4 index (based upon equatorial SSTs, 120–170° West^26^), the dipole mode index (DMI), and the SAM index (SAMI), respectively. To explore dependencies amongst climate modes, we used a wavelet-based principal components analysis of Niño3.4, DMI and SAMI to generate a single, combined index representing the climate system (the first wavelet principal component, wPC1) for 1982 to 2013, which is the longest period for which all three climate indices are available. Fluctuations in SAM explained 64% of the total variability amongst these three climate modes. The remaining 36% representing fluctuations in ENSO and IOD opens the possibility of amplification of one climate mode by the others, as occurred between ENSO and SAM during 2010 and 2011 along the east coast of Australia^38^.

**Figure 3:**
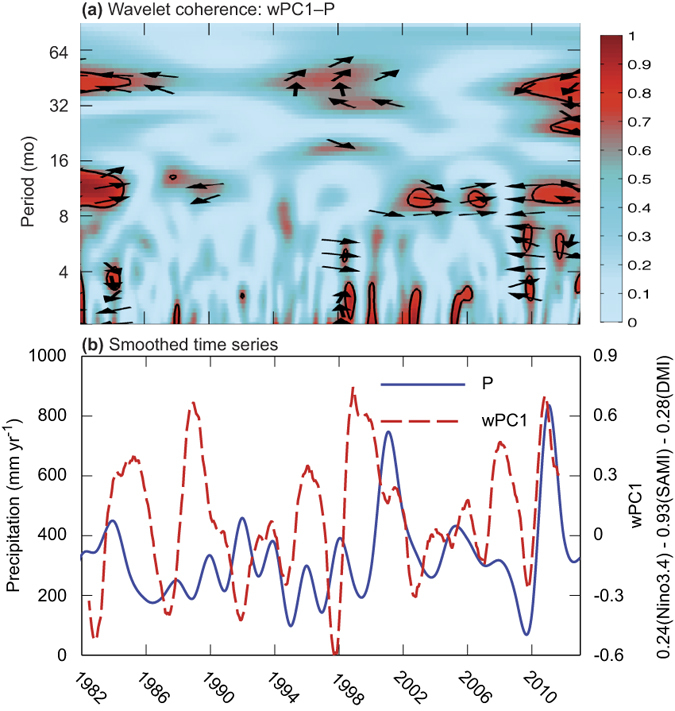
Relationship between local precipitation P (TGF,Fig. 5) and state of the coupled climate system (wPC1). (**a**) Normalised wavelet coherence between P and wPC1. Frequency-time coordinates of significant coherence are outlined. Arrows indicate phase (leftward: 180° out-of-phase; rightward: 0° in-phase) and degree of lead or lag (upward or downward) between wPC1 and P. The cone of influence was outside the domain of the plot. (**b**) Monthly P (solid blue line) and wPC1 (dashed red line), smoothed with a 12-month running average and scaled to annual values.

IOD is directly connected to ENSO and the tropical Atlantic Ocean *via* seasonal Walker circulations (i.e., tropical Hadley-type cells that propagate along an east–west axis from the convection centre^30^,^31^) (Fig. 1). Significant coherence between wPC1 and precipitation was observed at seasonal and annual timescales in the period 1999–2013 (Fig. 3). We propose that synchronisation amongst climate modes shifts with time, which is illustrated by the shift in coherence between climate modes and precipitation beginning in 1999 (Fig. 3) and which coincided with strengthening of the Walker circulation since the late 1990s^31^. It is when these shifting patterns of teleconnections between the Pacific and Indian Oceans are synchronised that we expect the combined effects of ENSO and IOD to be more extreme than either on its own.

The lagged, negative correlation between ENSO and IOD^30^ results in the tropospheric biennial oscillation (TBO), by which a strong Indian monsoon leads to a strong Australian monsoon and is then followed by a weak Indian monsoon^22^. The time lag is of crucial importance for establishing the oscillation, which arises from several feedback mechanisms (e.g., evaporation-wind feedback, SST-monsoon feedback, monsoon circulation-ocean thermocline variations)^21^. However, the TBO can be broken in the austral autumn, when Indian Ocean SSTs are altered by other influences (e.g., AMOC)^21^. As a consequence, sudden reversals in the climate system can lead to strong precipitation anomalies, such as those which occurred during 2009, a very dry year, when the correlation pattern between climate modes and precipitation changed phase (Fig. 3). After the TBO was broken during the austral autumn of 2009, a new phase was established by which heavy precipitation anomalies were sustained in central Australia during the period January 2010–March 2011 (Fig. 3).

## Extremes in weather and photosynthetic productivity across Australia

**Figure 4:**
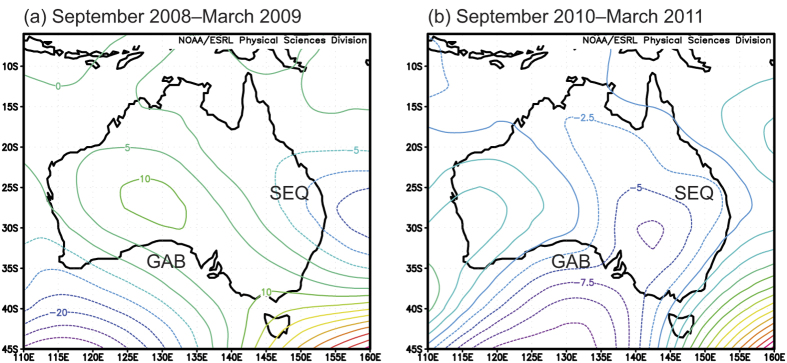
Seasonal weather patterns during (**a**) a very dry period (September 2008–March 2009) and (**b**) the land sink anomaly in Australia (September 2010–March 2011). Contours represent monthly average 500 hPa geopotential height above (solid lines) and below (dashed lines) the zonal average, in which negative values are indicative of low pressure and enhanced vorticity. The locations of southeast Queensland (SEQ) and the Great Australian Bight (GAB) are shown for reference. Maps were obtained from the NCEP/NCAR reanalysis project (monthly mean geopotential heights, http://www.esrl.noaa.gov/psd/cgi-bin/db_search/SearchMenus.pl).

**Figure 5:**
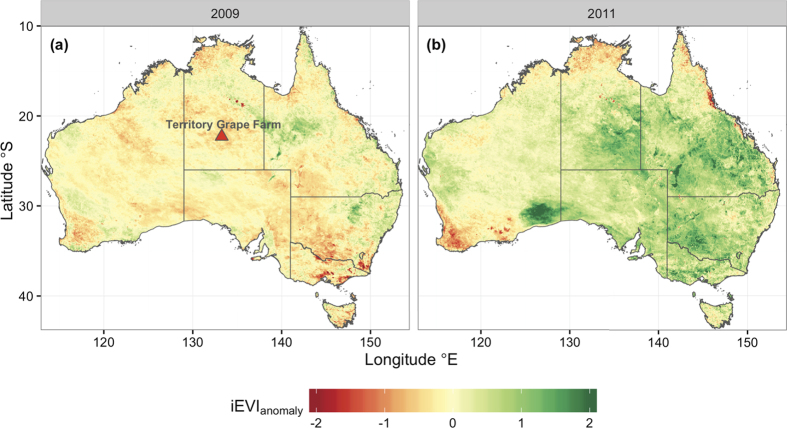
Biogeographic patterns in photosynthetic productivity over Australia during (**a**) a dry year (2008–2009) and (**b**) a wet year (2010–2011). Anomalies of annually integrated enhanced vegetation index (iEVI_anomaly_) were used as a surrogate for productivity. Large, positive values of iEVI_anomaly_ represent areas with a large C sink. The location of the Territory Grape Farm meteorological station (for precipitation in Figs 2 and 3) is indicated. Map was drawn using R version 3.1.2 (http://www.R-project.org/).

**Figure 6:**
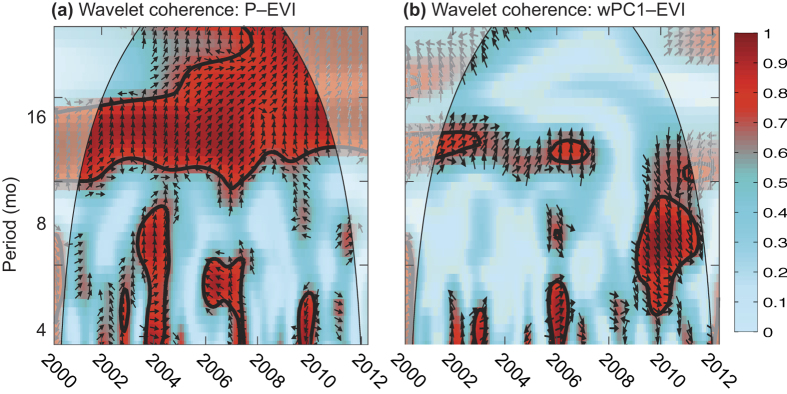
Normalised wavelet coherence between EVI and (**a**) P or (**b**) wPC1. The cone of influence is shown by the curved lines and shaded values. Tick marks on the x-axis are marked on the first day of the specified year.

To our knowledge, the correspondence between multiple climate modes, continental weather patterns, and productivity has not been demonstrated during the 2011 global land C sink anomaly or the preceding decade of drought, although several studies have investigated two of these three relationships on global and continental scales. By example in southeast Queensland (SEQ) during 2011, there was a clear relationship between La Niña individually and precipitation^28^, which can be seen in this study by the negative geopotential height anomalies along that portion of the east coast (Fig. 4b). Elsewhere in Australia, the interaction of ENSO, IOD and SAM affects fluctuations in precipitation, possibly more than any individual climate mode^2^,^16^,^23^. In this study, we found that the 2009 precipitation deficit, the 2011 precipitation surplus, and associated extreme fluctuations in productivity (Fig. 5) were correlated to synchronised shifts in the three climate modes (Figs 3 and 6) and linked weather patterns (Figs 4 and 6). The coupling of climate modes, and their interactive effects on weather patterns, explains why the world’s largest variations in precipitation, and potentially associated fluctuations in productivity, are found across the semi-arid regions of the Indian Ocean rim: northern Australia, Thar (India), and Somali (Africa)^39^.

The drought of 2008–2009 resulted in widespread reductions of the spatial extent of green, photosynthetic vegetation (Fig. 5a), representing the very small rates of photosynthesis that contributed to the global reduction in net primary production during that decade (2000–2009)^6^. Negative values of iEVI_anomaly_ in Fig. 5a illustrate widespread physiological inactivity by much of the vegetation (both grassland and woodland/shrubland) during drought, which was recently shown across the north-central and southeastern regions of the continent^40^,^41^,^42^. The spatial distribution of reduced productivity reflects weather patterns during the monsoon season (cf. Figs 4a and 5a), in which a high-pressure zone blocked development of the continental low over Western Australia and South Australia, whilst vorticity in the monsoon depression (Fig. 4a) was weakened by a positive IOD event. However, the weakened monsoon storm paths were evident in a strip of green that tracked across the north of the continent, following along geopotential contours toward a low-pressure zone over SEQ (cf. Figs 4a and 5a), which was present despite the near-neutral El Niño conditions during 2009 (Fig. 2b). Thus, breaking of the TBO in the austral spring preceding the 2008–2009 monsoon season was a consequence of a shift in the three climate modes, delivering dry weather patterns and a general failure in ecosystem productivity.

The greening of Australia in 2010–2011 was also not evenly distributed across the continent, but spatial variability in ecosystem productivity reflected weather patterns (cf. Figs 4b and 5b). Increased atmospheric vorticity followed a trough along a corridor from tropical northwestern Australia to the Great Australian Bight (GAB, Fig. 4b). This occurred by strengthening of the monsoon depression due to a strongly negative IOD event, combined with superimposition of the monsoon depression over the continental low, which can lead to intense vorticity in the upper atmosphere^43^. The resultant greenup within a very short timeframe (between 2008–2009 and 2010–2011) was indicative of the strong coherence between precipitation and EVI at an annual scale (Fig. 6a), reflecting the resilience of the native vegetation to hydrologic extremes (i.e., prone to shifts between no identifiable growing season in a dry year to extraordinary productivity in the following wet year)^40^,^41^,^42^,^44^,^45^,^46^. This resilience is a consequence of continental evolutionary and climatological history. The modern climate regime of Australia with its juxtaposition of very wet and dry years was established by the early Pleistocene (5 Million years before present), and the remaining vegetation are relics of historical rainforests that survived the semi-arid drying phase of the last 500,000 years^47^. Consequently, fluctuations between wet and dry periods can affect the sensitivity of stomatal conductance to vapour pressure deficit^48^. The resultant variations of inherent ecosystem water-use efficiency^49^ contributed to the very large C fluxes that were generated by vegetation during the C sink anomaly.

Precipitation is the primary driver of C uptake and emissions in moisture limited environments^50^,^51^. The strong correlation at an annual timescale between precipitation and productivity (precipitation–EVI coherence, where EVI is representative of photosynthetic productivity) illustrates the primary control of photosynthetic productivity by precipitation (Fig. 6a). At shorter timescales, precipitation was coherent with productivity during dry years (2003, 2004, 2007, 2009; cf. Figs 3a and 6a), but precipitation was only weakly correlated to productivity during wet periods (except 2005, Fig. 6a). Reductions in the correlation between precipitation and productivity during wet periods was a consequence of month-to-month variability in precipitation; for example, the coherence between precipitation and EVI was weak during 2001 (a very wet year) because of the uneven distribution of extremely wet and dry months during the wet season^52^. Similarly, correlations between climate modes and productivity (wPC1–EVI coherence) were not significant in 2001, a very wet year (Fig. 6b). Otherwise during the 2010 and 2011 wet years, wPC1–EVI coherence was stronger than for precipitation–EVI coherence (Fig. 6). The close similarities in wPC1–precipitation and precipitation–EVI coherences (cf. Figs 3a and 6b) suggests that synchronised fluctuations in ENSO, IOD and SAM affect both precipitation amount and frequency (i.e., inter-storm duration), which are more closely associated with fluctuations in the productivity of semi-arid regions than the amount of precipitation alone^53^.

## Conclusions

Variability in precipitation for the semi-arid environments of the Indian Ocean rim nations is enhanced relative to other semi-arid regions^39^ due to the impact of Indian Ocean SSTs on regional climate^29^,^54^. In this study, we demonstrate that the abnormally large fluctuations in precipitation experienced by Australia are not a result of any single climate mode but instead are due to periods of synchronisation amongst three climate modes: ENSO (equatorial Pacific Ocean), IOD (equatorial Indian Ocean) and SAM (Southern Ocean). This entirely novel finding has been speculated upon, but the method required to test this hypothesis has not been presented before now. Vegetation and ecosystems adapted to semi-arid conditions and unreliable precipitation generally show large variations in productivity^55^, switching between C cycle extremes depending upon the state of the climate^56^. In the semi-arid regions of Australia, precipitation extremes created by transient interactions amongst these climate modes have favoured vegetation that is very resilient to the juxtaposition between very dry and wet conditions^44^. Thus, large fluctuations in productivity across semi-arid Australia are the result of a long association over evolutionary timescales between vegetation and the fluctuating weather extremes that are induced by these interacting climate modes. Furthermore, it is the interaction of these climate modes that explains why Australia more than any other continent was at the heart of the 2011 global water and C sink anomalies^2^,^3^.

## Methods

### Climate datasets

Four datasets representing ocean–climate modes in the Pacific, Indian and Southern Oceans were used in this study. The southern oscillation index (SOI) represents the monthly pressure difference between Tahiti and Darwin, which was obtained from the National Climate Centre of the Australian Bureau of Meteorology for the period 1876–2013 (http://www.bom.gov.au). Thus, the monthly Niño 3.4 SST index was used for comparisons amongst climate modes. Niño 3.4 represents the SST gradient across the region bounded by 5° N–5° S and 120°–170° W^26^ and was obtained from NOAA/NCEP (USA, http://www.cpc.ncep.noaa.gov/data/indices/). The southern annular mode index (SAMI) is defined as the normalised monthly mean sea level pressure between 40° S and 70° S^34^ and was obtained from Nan and Li^57^ (http://ljp.lasg.ac.cn/dct/page/65572). The state of the IOD was estimated from the weekly SST dipole moment index (DMI) derived from NOAA OI SST version 2 (http://www.jamstec.go.jp/frcgc/research/d1/iod/HTML/Dipole%20Mode%20Index.html). The DMI was re-sampled to monthly average values to match the frequency of Niño3.4 and SAMI. Each index was normalised (−1 to 1) before evaluation of temporal correlations between climate indices and precipitation.

### NCEP synoptic maps

Seasonal weather patterns were identified from the 500 hPa geopotential heights, obtained from the NCEP/NCAR reanalysis project (http://www.esrl.noaa.gov/psd/cgi-bin/db_search/SearchMenus.pl)^58^. The height of the 500 hPa atmospheric pressure level is inversely related to vorticity. Data show the deviation from the zonal mean and represent the monthly average for September 2008–March 2009 (very dry year) or September 2010–March 2011 (very wet).

### Case study: central Australia

The historical weather station network of the Australian Bureau of Meteorology is sparsely distributed across the landscape due to the large area and small population. Only two meteorological stations in the Northern Territory have a record that is longer than 100 years (Alice Springs and Darwin)^59^. We chose a location intermediate between these two locations to represent an area that (1) is impacted by both continental low and the monsoon depression and (2) is known to have generated a large pulse of productivity during the 2011 land C sink anomaly^45^,^46^. This weather station (Territory Grape Farm, Fig. 5), which is located on Pine Hill Station near Ti Tree NT (200 km north of Alice Springs), has been in operation since 1987 (http://bom.gov.au).

To extend the meteorological record into the past and to fill missing values due to equipment failure, “patched point” meteorology from the SILO data drill (Queensland Department of Environment and Natural Resources, http://www.derm.qld.gov.au/silo) was used. In the data drill, missing daily records were constructed through spatial interpolation from the stations that were in operation at the time^60^. The representativeness of the patched point meteorology is shown in the reasonable comparison to satellite-derived precipitation from TRMM (Supplementary Fig. 3).

### Satellite measurements

Productivity by vegetation was inferred using the enhanced vegetation index (EVI) from the moderate resolution imaging spectroradiometer (MODIS) on board NASA’s Terra satellite^44^. EVI is a proxy for canopy “greenness”, which is an integrative composite property of green foliage, leaf chlorophyll content and canopy architecture^61^. In Australia, MODIS EVI is strongly correlated with gross primary productivity from eddy covariance flux towers across the wet-dry tropics and the semi-arid interior^62^. Approximately 14 years (2000–2014) of 16-day, 0.05° resolution MODIS EVI product (MOD13C1, Collection 5) were obtained from the NASA/USGS data depository (https://lpdaac.usgs.gov). Using the MOD13C1 quality control flags, only high-quality data were retained by excluding contaminated observations due to aerosols or clouds. Annually integrated EVI (iEVI) was used as a surrogate of productivity during an extreme drought year (September 2008–August 2009) and the land sink anomaly (September 2010–August 2011). VI anomalies during these years were calculated as the difference between iEVI in each respective year and mean iEVI during the entire record (2000–2014). A location near the Territory Grape Farm was identified for analysis of the correlation between EVI and climate or meteorological conditions. This location was chosen to avoid the effects of irrigated agriculture at the Territory Grape Farm on EVI. This ecosystem is characterised by an open Bloodwood (*Corymbia* spp.) savanna dominated by hummock grasses (*Triodia* spp.), which was a strong C source in the drought years following the 2011 land C sink anomaly^42^. EVI values were composited in a single 9 × 9 pixel centred on an eddy covariance tower at this OzFlux/Fluxnet site (Au-TTE).

Historical precipitation was obtained from the tropical rainfall-measuring mission (TRMM) to evaluate the representativeness of the precipitation data in central Australia. The TRMM data product (3B43-v7) for 1998–2013 was derived at a 0.25 × 0.25° spatial resolution by combining TRMM satellite data (from active radar and passive microwave retrievals), GOES-PI satellite data, and a global network of gauge data^63^,^64^.

### Wavelet statistics

Classical statistics often produce spurious results when applied to time series due to violation of fundamental assumptions (e.g., experimental variables that are neither independent nor identically distributed). Errors can arise due to undiagnosed auto-correlation and lagged cross-correlation, for which several methods have been developed to overcome statistical biases (e.g., auto-regressive analysis^65^, partial correlations^25^). To avoid these errors, spectral methods can be employed, but the global solutions resolved through Fourier transformation are only amenable to the most basic of statistical analyses. Consistent with the fundamental rules of Fourier transformations (admissibility, convolution, energy conservation)^66^, the wavelet transformation provides a linear analytical framework that is localised in time without violating statistical assumptions. By evaluating (co-)variances in the time series instead of averages, the resultant wavelet coefficients conform to the Central Limit Theorem and Reynolds averaging (the latter in the case of the discrete wavelet transformation)^67^.

Wavelet decomposition generates statistics that are based on relationships in time and frequency through dilation and translation of a mother wavelet Ψ:


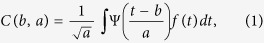


where *a* and *b* are the dilation (i.e., timescale) and translation (i.e., time) factors, *C*(*b*, *a*) is the wavelet coefficient at given time and scale, and *f*(*t*) is the time series against which Ψ is compared. Analysis of timescales was limited to 10 scales per octave *j* (*n* = 2^*j*^), but scales were not considered below the Nyquist frequency (i.e., one-half of the measurement frequency). More importantly, time and frequency scales can be evaluated individually and in combination, consistent with the rules of convolution. Thus, the coherence between SOI and precipitation was integrated with respect to frequency to generate a time series of correlations (cf. Fig. 2 and Supplementary Fig. 1).

When comparing multivariate time series, dependencies between “independent” drivers can also generate spurious results. In this case, the role of individual climate modes can be evaluated only when no interacting effects are identified (as shown in Supplementary Fig. 2). To construct a single composite climate index representative of the interaction term, a multi-scale and multivariate analysis was performed in Matlab R2013 using wavelet-based principal components analysis (wPCA). This is in contrast to the construction of the multivariate ENSO index (MEI), which uses a classical PCA (i.e., without a wavelet foundation) to incorporate various components of a single climate mode (e.g., atmospheric pressure, sea surface temperature, cloudiness)^37^. To improve processing time and to avoid cone of influence issues, wPCA was performed using a discrete wavelet transformation. A second-order symlet was chosen as a mother wavelet to 1) match the expected probability distribution function of the binomial-state climate index, 2) improve localisation in the frequency domain relative to the ‘Haar’ wavelet, and 3) reduce the effects of asymmetry in the higher-order Daubechies wavelets, upon which symlets are based. The first wavelet principal component (wPC1) was chosen to represent the coupled climate system (Niño3.4, DMI and SAMI).

Climate wPCA analysis was performed in three time periods: 1982–1998 (first half), 1982–2013 (full period) and 1999–2013 (second half). On the shorter timescales, SAMI dominated in the variability amongst the climate modes, whilst DMI fluctuations were independent of ENSO and SAM (Supplementary Table 1). However, dependencies amongst IOD, ENSO and SAM become apparent across the full period. The relationships between climate indices, precipitation and EVI were evaluated from the wavelet coherence between the coupled climate system and precipitation. Wavelet coherence analysis identifies the time periods and timescales at which two signals are correlated. The Morlet wavelet (defined as plane wave modified by a Guassian) was chosen for the basis function of the continuous wavelet decomposition for good localisation in the frequency domain and effectiveness in analysis of inhomogeneous signals^66^,^68^,^69^,^70^,^71^. The entire domain of monthly wPC1 values (i.e., 1982–2012; n = 363) was included in the analysis and all signals were padded. Padding with spectrally neutral (i.e., unvarying) values reduces the impact of edge effects within the cone of influence. Statistical significance of the coherence between climate indices and precipitation (α = 0.05) was inferred from a Monte Carlo comparison of the observed spectrum to a null model that was derived from an auto-regressive red noise spectrum^72^.

## Additional Information

**How to cite this article**: Cleverly, J. *et al.* The importance of interacting climate modes on Australia,s contribution to global carbon cycle extremes. *Sci. Rep.* **6**, 23113; doi: 10.1038/srep23113 (2016).

## Supplementary Material

Supplementary Information
