# Supplementary material for: The importance of interacting climate modes on Australia’s contribution to global carbon cycle extremes
Source: Sci Rep. 2016 Mar 15;6:23113. doi: 10.1038/srep23113 (PMC4791548; doi:10.1038/srep23113)
Supplement: Supplementary Information [file srep23113-s1.pdf]

Supplementary information

The importance of interacting climate modes on Australia's contribution to global carbon cycle extremes

James CLEVERLY*[1,2], Derek EAMUS[1,2], Qunying LUO[3], Natalia RESTREPO COUPE[3], Natascha KLJUN[4], Xuanlong MA[3], Cacilia EWENZ[5], Longhui LI[1], Qiang YU[1] and Alfredo HUETE[3]

[1] School of Life Sciences, University of Technology Sydney, PO Box 123, Broadway, NSW, 2007, Australia

[2] Australian Supersite Network, Terrestrial Ecosystem Research Network, University of Technology Sydney

[3] Climate Change Cluster, University of Technology Sydney

[4] Department of Geography, Swansea University, Singleton Park, Swansea SA2 8PP, UK

[5] Airborne Research Australia, Flinders University, PO Box 335, Salisbury, South Australia, 5106, Australia

*Correspondence: James.Cleverly@UTS.edu.au, +61 (02) 9514 8405, +61 (02) 9514 4079 (FAX)

Extended Data Table 1 | Wavelet-based principal components analysis statistics.

Date range	wPC1 (% of total)	wPC2 (% of total)
1982–1998	0.99 SAMI − 0.16 Niño3.4 − 0.03 DMI (70.5)	−0.16 SAMI − 0.95 Niño3.4 − 0.25 DMI (24.6)
1998–2013	−0.99 SAMI + 0.17 Niño3.4 + 0.04 DMI (80.2)	−0.17 SAMI − 0.98 Niño3.4 − 0.05 DMI (15.7)
1982–2013	−0.93 SAMI + 0.24 Niño3.4 − 0.28 DMI (>99)	

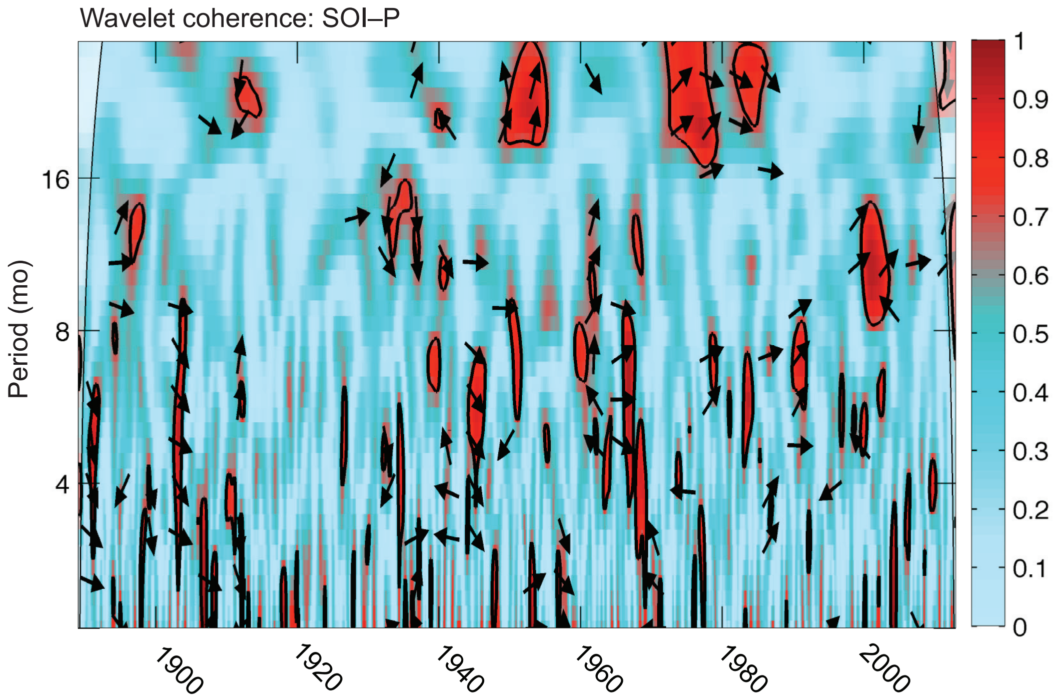

Supplementary Figure 1. Relationship between local precipitation P and the southern oscillation index (SOI). Frequency-time coordinates of significant coherence (i.e., squared correlation) are outlined. Arrows indicate phase (leftward: 180° out-of-phase; rightward: 0° in-phase) and degree of lead or lag (upward or downward) between SOI and P. The cone of influence is shown near the top corners of the plot.

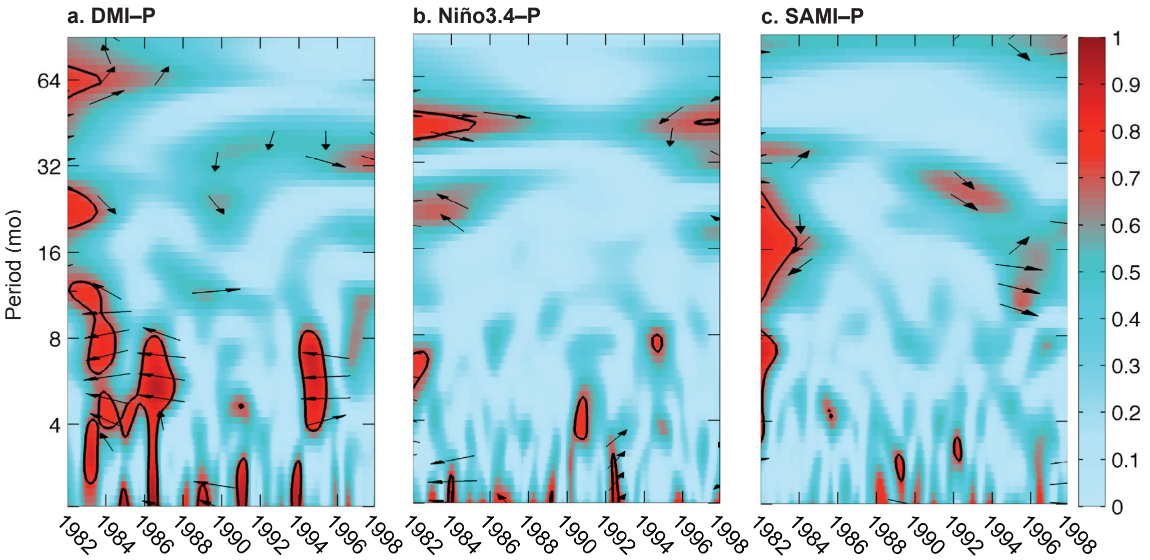

Supplementary Figure 2. Relationship between local precipitation P and climate modes: (a) dipole mode index (DMI), (b) Niño3.4 and (c) southern oscillation mode inde (SAMI). The time period shown when significant correlations between P and the combined climate index (wPC1) were absent (cf. Fig. 3). Frequency-time coordinates of significant coherence (i.e., squared correlation) are outlined. Arrows indicate phase (leftward: 180° out-of-phase; rightward: 0° in-phase) and degree of lead or lag (upward or downward) between Niño3.4 and P. The cone of influence was outside domain plot's domain.

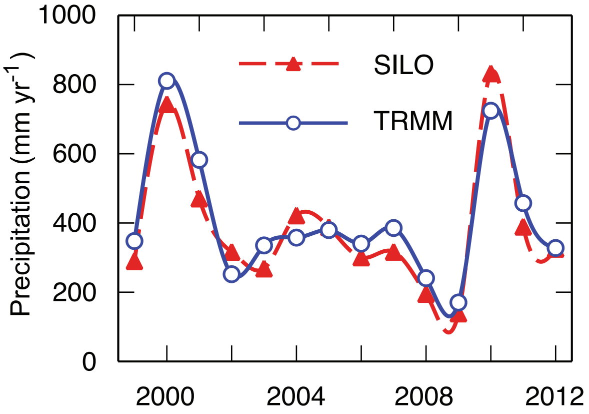

Supplementary Figure 3. Thirteen-year record of precipitation from SILO patched point observations (triangles and dashed red line) or TRMM satellite data (circles and solid blue line).
